# Insulin infusion sets associated with adverse events: strategies for improved diabetes education

**DOI:** 10.3389/fmed.2023.1275394

**Published:** 2023-11-29

**Authors:** Ana Lucia Domingues Neves, Luiz Eduardo Galvão Martins, Mônica Andrade Lima Gabbay, Paula Pascali, Tiago de Oliveira, Aldo Martinazzo, Sérgio Atala Dib, Dulce Elena Casarini, Sebastião Vagner Aredes, Fernanda Silva Tenorio, Tatiana Sousa Cunha

**Affiliations:** ^1^Postgraduate Program in Translational Medicine, Department of Medicine, Paulista School of Medicine, Federal University of São Paulo, São Paulo, Brazil; ^2^Department of Science and Technology, Institute of Science and Technology, Federal University of São Paulo, São Paulo, Brazil; ^3^Department of Medicine, Paulista School of Medicine, Federal University of São Paulo, São Paulo, Brazil; ^4^Diabetes Center Insulin Pump Ambulatory, Federal University of São Paulo, São Paulo, Brazil; ^5^DeltaLife, São José dos Campos, Brazil

**Keywords:** insulin infusion system, technology, diabetes mellitus, type 1, catheters, cannula

## Abstract

**Introduction:**

Insulin Infusion Sets (IIS) play a crucial role in ensuring the safe delivery of insulin through a Continuous Subcutaneous Insulin Infusion (CSII) for individuals with Type 1 Diabetes (T1D). Recent advancements in therapy have highlighted the need to address issues such as unexplained hyperglycemia and IIS occlusion.

**Objective:**

To investigate the adverse events (AEs) associated with IIS that impact the treatment of T1D, with a specific focus on promoting effective educational practices.

**Methods:**

One hundred and eighteen patients under treatment at the Diabetes Center Insulin Pump Ambulatory, Federal University of São Paulo responded to a semi-structured questionnaire. Over 6 months, a nurse researcher interviewed them via video calls.

**Results:**

Catheter-related adverse events (AEs) included catheter knots, folding, and accidental traction. AEs associated with cannula use were mainly related to cannula fixation adhesive, insulin leakage, bleeding episodes, and skin problems. The cannula patch tends to detach easily in hot conditions or when used for more than 3 days, leading to local itching. Adhesive glue can cause redness and pain. Insulin leakage typically occurs after the catheter disconnects from the cannula, accidental cannula traction, or beneath the cannula patch. Bleeding has been reported inside the cannula or at the insertion site, resulting in local pain and, in some cases, obstruction of insulin flow. When accidental cannula traction occurs, it is recommended to replace the entire IIS system. In situations involving bleeding, leakage, insulin odor, or unsuccessful attempts to correct hyperglycemic episodes with a “bolus” of insulin, it is advisable to change the IIS system and evaluate appropriate techniques for handling and infusing the device. Moreover, regular inspections of the device and reservoir/cartridge are essential.

**Conclusion:**

Serious AEs can occur even in cases where the occlusion alarm is not activated, leading to interruptions in insulin flow. Conversely, in less severe situations, alarm activation can occur even in the absence of insulin flow interruption. Accidental catheter traction and catheter bending are commonly encountered in everyday situations, while issues related to the cannula directly affect blood glucose levels. AEs related to the IIS cannula often involve insulin leakage into the skin, bleeding, and skin events attributed to adhesive issues with the cannula.

## 1 Introduction

The treatment of Type 1 Diabetes Mellitus (T1D) can be performed through continuous subcutaneous insulin infusion (CSII) or multiple daily injections (MDI) (1). These methods are effective and safe for implementing intensive management of Diabetes Mellitus (DM) (2, 3).

We describe CSII as an evolutionary form of insulin administration that effectively maintains glycemic levels, offers flexibility to patients, and induces fewer instances of hypoglycemia. The Diabetes Control Complications Trial (DCCT) study (1) demonstrated that intensive treatment with CSII reduces the incidence and progression of microvascular complications when compared to conventional treatment with MDI (3–6).

There is evidence from reviews and meta-analyses showing that the use of CSII reduces the frequency and duration of hypoglycemic events, decreases episodes of recurrent diabetic ketoacidosis, and improves glycemic control and quality of life compared to conventional treatment with MDI (7–13). Moreover, for young children, CSII may be the only option, as their daily insulin needs are very low, requiring doses lower than 0.5 U, which is impractical with MDI therapy (14).

It is well-established that CSII is a critical medical device that introduces new challenges and vulnerabilities due to the inherent risk of technological failures (15). The safety and efficacy of CSII use are highly dependent on appropriate selection of the patient, their level of diabetes education, adherence to therapeutic recommendations, and the technical proficiency and competence of the multidisciplinary team responsible for their care (16). Thus, the use of CSII should be based on strict criteria and the indication should be exclusively for eligible patients. It is known that approximately 15% of patients with T1D have an absolute indication for its use (17).

Conceptually, adverse events (AEs) can be categorized into “nonmetabolic AEs” related to events in the catheter infusion set, issues with the device itself (such as software or screen problems, keyboard issues, battery or component failures, insulin leakage, or water damage), or skin complications. “Metabolic AEs” result from diabetic ketoacidosis and severe hypoglycemia, among other reasons, often tied to failures in the infusion set or errors in the calculation of the “bolus” (18).

According to the Food and Drug Administration (FDA) Code of Federal Regulations (CFR) 21, part 803 (19), the primary language for defining an AE is as follows:

Caused or contributed means that death or serious injury was or may have been attributed to a medical device, or that a medical device was or may have been a factor in death or serious injury, including events occurring as a result of (1) Failure, (2) Malfunction, (3) Improper or inadequate design, (4) Manufacture, (5) Labeling, or (6) User error.

Reliable insulin delivery depends directly on the proper functioning of the IIS, which includes the cannula and catheter associated with fluid transfer (20, 21). Recent advances in IIS technology emphasize the importance of discussing aspects related to these components of CSII therapy, as all users of the device rely on the IIS for the predictable delivery of insulin to the subcutaneous tissue (22).

Issues related to IIS accounted for the majority of CSII recalls by the US FDA (2). Various studies (4, 15, 23) have examined the frequency and types of adverse events (AEs) associated with CSII, including blockages, bent catheters, and insertion site reactions such as irritation and areas of infection, all of which can compromise metabolic control. To minimize these AEs, manufacturers have historically recommended changing the catheter every 2–3 days (15, 18, 23–25).

Recent studies have examined potential causes of AEs associated with CSII models, including the MiniMed™ 670G, MiniMed™ 630G, Omnipod_®_, Omnipod DASH_®_, and t:slim X2™. These investigations utilized data from the FDA Manufacturer and User Facility Device Experience (MAUDE) database. Out of the 2,429 AEs reported, approximately 8% were linked to issues with the infusion set or site (26).

Problems related to the IIS, such as displacement, cannula blockage, skin reactions, the cannula being placed in unhealthy tissue (e.g., areas of Lipohypertrophy), and unpredictable variations in insulin absorption, significantly impact therapy. Additionally, uninterrupted insulin delivery can be affected by factors such as the choice of cannula insertion site, interstitial pressure, and occlusion by cell debris or insulin. The precise causes of insulin delivery inaccuracies related to IISs are multifactorial and not yet well understood (20, 21). Early detection of these factors is crucial to minimize the risk of prolonged hyperglycemia, especially in automatic insulin administration methods such as CSII therapy (27).

Given the limited information available regarding AEs linked to CSII and the substantial incidence of these events affecting the treatment of T1D, this study aims to describe the factors associated with IIS that significantly influence disease management. The primary objective is to facilitate the development of clinical practice protocols and patient guidance by presenting these factors, with a particular emphasis on promoting effective educational practices.

## 2 Materials and methods

### 2.1 Study stages

In this section, we present an overview of the proposed methodology, which is divided into four main steps, highlighting their sequence and iteration.

The study was conducted in four phases. In Phase I, a literature review was performed using databases such as Medline via PubMed, Lilacs, Science Direct, and Scielo to identify evidence related to prevalent AEs associated with the use of CSII systems. English descriptors and their Portuguese equivalents, such as “Diabetes Mellitus, Type 1,” “Health,” “Insulin,” “Insulin Infusion Systems,” and “Adverse Event,” were employed. These descriptors were combined using appropriate Boolean operators. No restrictions were placed on the year or language of publication. Additionally, manual searches were conducted by exploring the reference lists in the identified documents. During this stage, the entire process of searching, selecting, and extracting data from the articles was carried out in pairs. Data were organized into extraction tables containing basic information, including the title, authors, affiliation, country, journal title, and publication date. Studies involving pregnant women with T2D or the use of devices in a hospital environment, as well as studies with methodological inconsistencies, were excluded.

Subsequently, full-text articles were obtained, and in cases of uncertainty, consensus meetings were held with mentors to determine whether to include or exclude an article. Finally, AEs associated with the device, IIS, and device battery described in the literature were categorized.

Phase II marked the beginning of data collection, aiming to explore reports from patients followed at the Diabetes Center. This phase provided a general overview of prevalent AEs associated with the device and gathered essential data for the future description of safety requirements for the research group’s low-cost CSII prototype.

Before beginning this stage, the researcher established telephone contact with each research participant and their family members to explain the study, apply the Informed Consent Form, and clarify any potential doubts. Subsequently, the Data Collection Instrument was administered through semi-structured interviews conducted via video calls.

The data collection instrument was adapted from the “*Survey on Complications of Insulin Pump Therapy*,” mentioned in the publication “Nonmetabolic Complications of Continuous Subcutaneous Insulin Infusion: A Patient Survey” (28), and the copyright was kindly provided by the author John C. Pickup via email. This instrument was validated by the project’s interdisciplinary team and consisted of three sections: S1: Sociodemographic aspects; S2: General information about the disease and the device used for treatment; and S3: Data related to the insulin infusion set.

At the conclusion of Phase II, the data were organized into three main categories: “General data on the disease and CSII,” “Insulin Infusion Set Data,” and finally, “CSII Usability Data: User Interface.”

Phase III of the study involved the preparation of a document entitled “Description of Adverse Events associated with the use of CSII: contributions to the Safety Requirements of a low-cost medical device.” In this document, the AEs were organized into three main categories: “General Data on the disease and CSII,” “Insulin Infusion Set Data,” and finally, “CSII Usability Data: User Interface.”

In Phase IV of the study, the prevalence of identified AEs was calculated using simple arithmetic means.

### 2.2 Data collection

The present study was conducted at the Diabetes Center Insulin Pump Ambulatory, Federal University of São Paulo, following the guidelines of the Institutional Research Ethics Committee (REC) (reference no. REC/UNIFESP 1242/2019) and the principles of the Helsinki Declaration. A total of 189 patients who were undergoing treatment for T1D at the study site were invited to participate.

After excluding those who declined and those who did not respond, a total of 118 patients (with a participation rate of 63%), including children, adolescents, and adults, who had been using any brand and model of CSII for at least 6 months, participated in the study. Each participant provided electronic informed consent, and data collection was conducted remotely over 6 months due to the COVID-19 pandemic.

The data collection instrument consisted of a semi-structured interview conducted by a researcher nurse through video calls (29). We developed a questionnaire comprising three sections, which underwent content validation by the projects’ interdisciplinary team. The first section included sociodemographic aspects such as age, sex, city of origin, education, and ethnicity. The second section focused on general information about T1D, and the device being used, including the date of diabetes diagnosis, initiation of CSII treatment, number and reasons for device change, and device manufacturer/model, among other details. The last section included general data related to the IIS, as described below.

1. What are the dimensions of the cannula and catheter used?
2. How frequently do you replace the IIS cannula and catheter?
3. What factors contribute to the recommended change of the cannula after approximately 3 days, as advised by the healthcare facility responsible for the treatment of T1D?
4. How often and under what conditions are adverse events associated with accidental catheter traction, catheter knotting, and catheter bending observed?
5. Were there any disruptions to basal/bolus programming and alarm systems during the occurrence of the aforementioned adverse events?
6. Have there been any reported complications at the site of cannula insertion, such as local bleeding or wounds, poor adhesive adhesion, signs of skin infection or allergies, or insulin leakage, among others?
7. How often and under what circumstances has insulin leakage occurred at the site where the catheter is connected to the insulin reservoir?

### 2.3 Context of the use of CSIIs at the time of data collection

The CSII system manufactured by Medtronic allows for connectivity with continuous glucose monitoring (CGM) sensors through radio frequency, except for the 715 model.

The CGM system comprises the Enlite_®_ glucose sensor and the Guardian_®_ Link transmitter, which capture real-time data from the sensor and transmit them to the CSII device. For optimal performance of the Medtronic device, it is recommended to use Energizer_®_ batteries, specifically the AAA alkaline model (24).

The Roche system, notably the *Accu-Chek Spirit Combo*, is a medical device designed to simulate the functionality of a healthy pancreas. It consists of two primary components: the CSII and a glucometer referred to as the control unit. The CSII is responsible for delivering insulin according to the specific requirements of the patient, while the control unit is equipped with Bluetooth technology for wireless connectivity with the CSII. Additionally, the glucometer measures the patient’s blood glucose levels and calculates suggested insulin dosages for precise administration at each relevant moment.

The Secretary of Health in São Paulo adheres to rigorous criteria when approving local recommendations and authorizing the use of CSII therapy for patients. Generally, this therapy is indicated for patients who experience severe hypoglycemia (provided they are sufficiently insulinized through MDI), as well as those who suffer from nocturnal or asymptomatic hypoglycemia, particularly among children under 6 years of age. These very young children not only face a heightened risk of hypoglycemia but also exhibit substantial glycemic variability and require low-dose insulin treatment. In cases of severe hypoglycemia among very young children, the use of a sensor manufactured by Medtronic is recommended.

In this study, all participants using the Roche system were equipped with the Accu-Chek Spirit Combo, which includes the device and the glucometer, along with all necessary supplies such as cannulas and catheters. The integration of the glucometer into the Roche system enables patients to access real-time glucose readings, simplifying the process of precise insulin dosing. This functionality significantly enhances the management of T1D and leads to greater overall clinical stability.

## 3 Results

### 3.1 Characterization of the socio-demographic, disease profile, and device

Out of the total number of participants in the study (118), 71 were women and 47 were men. Among the participants, 51 belonged to the pediatric age group while 67 were adults, with mean ages *x̄* = 10 years and 29 years, respectively. The majority of the participants, specifically 88 individuals, identified themselves as belonging to a white racial background. Additionally, 70 participants resided in the city of São Paulo. Lastly, it is worth noting that 39 participants had either completed their postgraduate studies or were currently pursuing higher education.

Regarding the moment of T1D diagnosis, we observed the following trends. In the pediatric population and adolescents aged 2–18 years, of the 51 patients, 8 were diagnosed during the 1990s and 43 were diagnosed during the 2000s. Among adults, who included 67 patients aged 19–55 years, 3 were diagnosed in 1984 and 1989, 19 were diagnosed between 1990 and 1999, and 45 were diagnosed between 2000 and 2016.

The time interval between the T1D diagnosis and the commencement of CSII therapy varied depending on the age group. For pediatric patients, the interval ranged from 1 to 15 years, while for adults, it spanned from 7 to 37 years. In terms of the average duration of device usage, the study found that pediatric patients utilized the device for an average of 5 years, whereas adult patients used it for an average of 9 years. According to Table 1, the most commonly used device was the CSII Roche - Accu-Chek Spirit Combo. Several Medtronic models were also used, and one patient in the study was using a Tandem_®_ Diabetes Care device. It is noteworthy that out of the four interviewees using Medtronic devices who were unable to specify the model being used, three were mothers of children between 3 and 5 years old, and the fourth patient was 29 years old and had been using the device for 9 years.

**Table 1:** Distribution of patients using different models and brands of CSII at the Diabetes Center Insulin Pump Ambulatory, Federal University of São Paulo (São Paulo, Brazil, 2021).

CSII manufacturer and model	Patient number
Roche® – Accu-Chek Spirit Combo	67
Medtronic® – MiniMed Paradigm Veo 754	29
Medtronic® – MiniMed Paradigm 715	09
Medtronic® – MiniMed Paradigm 722	05
Medtronic® (model unknown)	04
Medtronic® – MiniMed 640G	03
Tandem® Diabetes Care	01
Total	118

### 3.2 Characterization of the sizes and change frequencies of IIS, and proposals for educational actions

The frequency of replacing the IIS cannula and catheter varied among the patients in the study. Out of the 118 participants, 89 individuals reported replacing both the cannula and catheter at the same frequency of use. Within this group, 60 participants reported changing them up to every 3 days, while 29 participants reported changing them every 3–7 days (Table 2). In contrast, the remaining 29 individuals reported replacing the cannula and catheter at different frequencies (as shown in Table 3).

**Table 2:** Frequency of IIS cannula and catheter change in patients monitored at the Diabetes Center Insulin Pump Ambulatory, Federal University of São Paulo–change of both the cannula and catheter at the same frequency of use (São Paulo, Brazil, 2021).

Same frequency (days) of change of IIS cannula and catheter (*n* = 89)			
Cannula and catheter use days	Patients number		Device manufacturer
2	5	1	Tandem® Diabetes Care
		3	Medtronic® – MiniMed Paradigm Veo 754
		1	Roche® – Accu-Chek Spirit Combo
3	55	24	Roche® – Accu-Chek Spirit Combo
		16	Medtronic® – MiniMed Paradigm Veo 754
		6	Medtronic® – MiniMed Paradigm 715
		3	Medtronic® – MiniMed 640G
		3	Medtronic® – Paradigm 722
		3	Medtronic® model unknown
4	5	2	Roche® – Accu-Chek Spirit Combo
		1	Medtronic® – Paradigm 722
		2	MiniMed Paradigm® Veo 754
5	2	2	Roche® – Accu-Chek Spirit Combo
3–4	14	8	Roche® – Accu-Chek Spirit Combo
		2	Medtronic® – MiniMed Paradigm 715
		4	Medtronic® – MiniMed Paradigm Veo 754
3–5	2	1	Roche® – Accu-Chek Spirit Combo
		1	Medtronic® – MiniMed Paradigm Veo 754
3–6	1	1	Medtronic® – MiniMed Paradigm Veo 754
3–7	2	2	Roche® – Accu-Chek Spirit Combo
4–5	3	1	Roche® – Accu-Chek Spirit Combo
		1	Medtronic® – MiniMed Paradigm 715
		1	Medtronic® – MiniMed Paradigm Veo 754

**Table 3:** Frequency of IIS cannula and catheter change in patients monitored at the Diabetes Center Insulin Pump Ambulatory, Federal University of São Paulo –change of cannula and catheter at different frequencies (São Paulo, Brazil, 2021).

Different frequencies (days) of change IIS cannula and catheter (*n* = 29)				
Cannula use days	Catheter use days	Patients number		Device manufacturer
2	4	2	2	Roche® - Accu-Chek Spirit Combo
3	3–5 (1)	17	14 1 1 1	Roche® - Accu-Chek Spirit Combo Medtronic® model unknown Medtronic® - Paradigm 722 Medtronic® - MiniMed Paradigm Veo 754
	4 (1)			
	4–5 (1)			
	5 (1)			
	6 (8)			
	7 (3)			
	Variable (2)			
4	5–6	3	3	Roche® - Accu-Chek Spirit Combo
	6			
	7–10			
3–4	5 (2)	5	5	Roche® - Accu-Chek Spirit Combo
	Variable (1)			
	6 (1)			
	7 (1)			
3–5	5–7 (1)	2	2	Roche® - Accu-Chek Spirit Combo
	Variable (1)			

The cannula size and catheter length reported by the patients at the time of the study are shown in Table 4.

**Table 4:** Characterization of the cannula size and catheter length.

Brand and model of device	Size cannula (mm)	*n*	Brand and model of device	Catheter length (cm)	*n*
Medtronic® (model unknown)	5 mm	2	(22) Medtronic® - MiniMed Paradigm Veo 754	60 cm	90
Medtronic® – MiniMed Paradigm Veo 754			(6) Medtronic® - MiniMed Paradigm 715		
Medtronic® (model unknown)	6 mm	27	(3) Medtronic® - MiniMed 640G		
Roche® – Accu-Chek Spirit Combo			(4) Medtronic® - Paradigm 722		
Medtronic® – MiniMed 640G			(2) Medtronic® (model unknown)		
Medtronic® – MiniMed Paradigm 715			(52) Roche® - Accu-Chek Spirit Combo		
Medtronic® – MiniMed Paradigm Veo 754			(1) Tandem® Diabetes Care		
Roche® – Accu-Chek Spirit Combo	8 mm	18	(8) Roche® - Accu-Chek Spirit Combo	30 cm	18
Roche® – Accu-Chek Spirit Combo	8.5 mm	1	(3) Medtronic® - MiniMed Paradigm 715		
Medtronic® (model unknown)	9 mm	26	(6) Medtronic® - MiniMed Paradigm Veo 754		
Medtronic® – Paradigm 722			(1) Medtronic® (model unknown)		
Medtronic® – MiniMed 640G			(7) Roche® - Accu-Chek Spirit Combo	size unknown	10
Medtronic® – MiniMed Paradigm Veo 754			(1) Medtronic® (model unknown)		
Medtronic® – MiniMed Paradigm 715			(1) Medtronic® - Paradigm® 722		
Roche® – Accu-Chek Spirit Combo	10 mm	30	(1) Medtronic® - MiniMed Paradigm Veo 754		
Roche® – Accu-Chek Spirit Combo	13 mm	3			
Tandem® Diabetes Care					
Roche® – Accu-Chek Spirit Combo	17 mm	3			
Medtronic® – MiniMed Paradigm Veo 754	Size unknown or do not remember	8			
che® – Accu-Chek Spirit Combo					

Diabetes Center Insulin Pump Ambulatory, Federal University of São Paulo (São Paulo, Brazil, 2021).

The recommendations below aim to ensure close monitoring of glycemic control, effectively address hyperglycemic episodes, and take appropriate actions if blood glucose levels remain high. Additionally, monitoring device disconnects and suspensions can help identify any issues related to the pump’s functionality. It is important to address any factors that may delay the IIS change and provide educational support to help patients overcome these challenges. Finally, engaging in open discussions with patients about their concerns allows for personalized treatment planning and helps establish realistic goals.

The recommendations regarding changing the IIS for the patients are as follows:

1. When uncertain about the date of the last cannula and catheter change, it is advised to change the entire IIS.
2. Consider changing the catheter if you notice changes in your glycemic profile, such as inadequate correction or ineffective “meal bolus,” as well as correcting pre-meal hyperglycemia.
3. It is recommended to increase your glycemic monitoring if the duration between IIS changes exceeds the recommended 3 days set by the study site team.

The Healthcare Team/Educators in diabetes follow these recommendations:

1. Monitor the glycemic profile while using the current device settings. In the case of hyperglycemic episodes, after administering two corrections, if blood glucose levels remain high, administer insulin with a pen and change the entire IIS.
2. Observe device disconnects/suspend patterns, and whenever there are changes in settings, administer insulin with a pen and change the entire IIS.
3. Pay attention to factors that may delay the IIS change, such as financial or motivational issues, and take educational actions to address these concerns.
4. Initiate a dialogue with the patient regarding their concerns about the IIS change technique to develop an individualized and realistic treatment plan.

The following tables (Tables 5, 6) provide a comprehensive overview of the reasons given for various scenarios regarding cannula and IIS catheter change.

**Table 5:** Characterization of reasons reported for cannula and catheter change within 3 days or more–change at the same frequency.

Same frequency (days) of use of IIS cannula and catheter	Reason	*n*
Up to 3 days	Accidental catheter traction: these individuals experienced unintentional pulling on the IIS, leading to the need for an early change	16
	Detachment of adhesive from the cannula occurred earlier than expected, without any apparent reason, on the leg or arm	8
	Detachment of adhesive due to heat: the adhesive on the cannula detached earlier than expected due to high temperatures	2
	Detachment of adhesive due to humidity: the adhesive on the cannula detached earlier than expected due to high humidity levels	4
	Insulin leakage into the skin: these individuals experienced insulin leakage from the IIS, requiring an early change of the IIS	2
	High glycemic values without improvement: these patients had persistently elevated blood glucose levels that did not improve even after changing the IIS. One case involved hospitalization in the Intensive Care Unit due to ketoacidosis, and another was reported by a mother who noticed her child’s blood glucose always increasing before the 3-day mark	22
	Persistently high glycemic values after catheter change: these patients had persistently elevated blood glucose levels even after changing the catheter, leading to the decision to change the entire IIS	2
	One patient reported the accumulation of insulin inside the cannula, prompting an early change	1
	Catheter bending with insulin flow interruption (2 patients): the catheter became kinked, disrupting insulin flow	2
	Cannula knotting under the skin (10 patients): the cannula formed a knot or loop under the skin, requiring an early change of the IIS	10
	Catheter macerated by dog bite (1 patient): the catheter was damaged by a dog bite, necessitating immediate change	1
	Abdominal Lipohypertrophy (1 patient): this patient had abdominal Lipohypertrophy, a condition characterized by the breakdown of fatty tissue, requiring an early change of the IIS	1
	Repeated occlusion messages (1 patient): this patient received repeated occlusion messages from the device, leading to the decision for an early change	1
	Cannula insertion site complications: there were various complications reported at the cannula insertion site, including bleeding (2 patients), pain (4 patients), swollen spots (1 patient), itching (1 patient), burning (1 patient), signs of inflammation (1 patient), and local discomfort (2 patients)	12
	Sun exposure affecting insulin effectiveness (1 patient): patients reported that prolonged sun exposure altered the effectiveness of insulin, necessitating an early change of the IIS.	1
	Inadequate cannula insertion due to a problem with the “applicator”: patient experienced difficulties with the “applicator,” leading to improper cannula placement and the need for an early change	1
	Concomitant change of supplies and insulin reservoir due to excessive insulin use: these patients reported excessive insulin use, requiring a simultaneous change of both the IIS and insulin reservoir	5
More than 3 days	Waiting for the same day to change the insulin reservoir (20 patients): some patients preferred to change both the IIS and insulin reservoir on the same day, especially if there was still insulin remaining in the reservoir due to consuming less food (3 patients) or if there was no itching or discomfort at the infusion site	20
	Lack of supplies influenced some patients’ decision to change the cannula and catheter less frequently: patients reported cases where they had limited access to the inputs and therefore chose to use the components for a longer period (up to 15 days). They believed that extending the time of use without compromising health or therapy was a cost-effective approach	15
	Forgetting (12 patients): these individuals reported forgetting to change the IIS at the recommended interval. Some of them experienced burning and redness at the infusion site due to extended use	12
	No local skin discomfort: patients reported extending the IIS change because they did not experience discomfort or irritation of the skin (cannula insertion site), and the components still worked effectively even after a long period of use. One patient reported that when the cannula was in the leg, there was greater sensitivity and local itching after the recommended period	4
	Stable blood glucose levels: these individuals delayed changing their IIS for a maximum of 5 days, as their blood glucose levels remained stable	4
	Exceptional circumstances: one patient reported that prolonged use was due to the hospitalization of his caregiver’s father, which affected his ability to change the IIS. Another reported waiting for the main caregiver to return from work before changing the IIS. Another two patients prolonged the use of the IIS when they were traveling or away from home	4
	Night shift schedule or late arrival at home: patients cited working hours or getting home late as reasons for not changing the IIS within the recommended range	3
	Missed message or lack of awareness: patients reported that their mother did not hear the message issued by the device or did not heed the child’s warning about the need for IIS change	2

Diabetes Center Insulin Pump Ambulatory, Federal University of São Paulo (São Paulo, 2021).

**Table 6:** Characterization of reasons reported for cannula and catheter change within 3 days or more–change at different frequencies.

Different frequency of use of IIS cannula and catheter	Reason	*n*
Up to 3 days	Early externalization of the cannula during vigorous physical activity	1
	Bleeding at the cannula insertion site	1
	Undue leakage of insulin at the cannula insertion site	2
	Insulin catheter obstruction	1
	Insulin catheter occlusion message	1
	Cannula bending	1
	Inflammation at the cannula insertion site	3
	High blood glucose levels	3
	Accidental cannula traction	5
	Pain at the cannula insertion site	6
	Pain at the cannula insertion site (usually in the flank)	1
	Redness	2
	Detachment of the patch from the skin in the summer	1
More than 3 days	Lack of supplies	4
	Following the routine guided by the doctor at the basic health unit or a member of the health team	2
	Changing the catheter along with the reservoir when the insulin runs out	24
	Working night shifts on the day of the IIS change	1
	Forgetting	3
	Following the guidance received when installing the CSII 4 years ago	1
	The belief that the catheter is still functioning properly	1
	Inability to remember the date of the last change	1
	Perceiving no risk of IIS obstruction	1

Diabetes Center Insulin Pump Ambulatory, Federal University of São Paulo (São Paulo, 2021).

### 3.3 AEs associated with IIS cannula and catheter

Catheter- and cannula-related AEs can be classified as cutaneous and subcutaneous complications and technical issues.

#### 3.3.1 Catheter

Among the patients included in the study, three relevant AEs directly involving the IIS catheter were identified (Tables 7–9). These AEs are described below, along with their respective characteristics and frequencies of occurrence.

- Occurrence of accidental catheter traction: this AE rarely occurred among 62 patients and involved the unintended removal or dislodgement of the IIS catheter. The occurrences of this AE were distributed as follows:
  − *N* = 11 patients experienced the event, but no explanation was provided for its occurrence.
  − *N* = 30 patients attributed the AE to physical contact with another person, such as during play, in a water park, while getting dressed, falling, sleeping, or accidental contact with objects like doors, tables, chairs, or zippers. These incidents resulted in either partial or total traction of the catheter.

− *N* = 18 patients reported that the occurrence of accidental catheter traction was more frequent depending on the positioning site of the cannula. It was more commonly reported in the leg (10 cases), arm (5 cases), buttocks (2 cases), or belly (1 case).

− *N* = 1 patient reported an accidental catheter traction that resulted.

in the rupture of the catheter at the cannula-catheter intersection.

− *N* = 1 patient attributed the occurrence of the AE to the absence of a clip to secure the device on clothing, especially when fixed on the belly or leg.

− *N* = 1 patient experienced accidental catheter traction due to inadvertently pressing the cannula “button.”

− In three exceptional cases (*N* = 3), accidental traction involved both the cannula and catheter, such as when the catheter was caught in a car door or during use on the leg.

**Table 7:** Characterization of the type and frequency of “accidental catheter traction” and proposals for educational actions.

Accidental catheter traction (*n* = 70)			
AEs	*n*	Frequency	Actions
Dislodged catheter (with no explanation for its occurrence)	11	Rarely	Patient Actions: For patients, it is advisable to change the IIS when either total or partial displacement of the catheter is confirmed. It is important to observe which sites on the body experience less catheter friction during daily activities or professional life to minimize discomfort or the risk of dislodgement. By identifying optimal sites for placement, patients can enhance their overall experience with the IIS and reduce potential complications. Healthcare Team/Educator Actions: For the health team or diabetes educator, it is crucial to evaluate, together with the patient, adapting the IIS insertion site to their profile. This involves considering factors such as body shape, lifestyle, and preferences. The team should also provide instructions to the patient on site rotation care, ensuring that different areas of the body are used for IIS placement to avoid overuse of a specific site. Additionally, they should educate the patient on preventive measures, such as protecting the infusion line with suitable adhesive tape to prevent accidental dislodgement or damage during activities.
Dislodged catheter during daily living activities and in physical contact With another person: When playing In the water park When getting dressed When falling When sleeping When catching a catheter in the door, table, chair, or in the zipper of the pants (with total traction or partial)	30	Rarely	
Catheter dislodgement directly related to the cannula placement site, more frequently in the buttocks (*n* = 2), leg (*n* = 10), arm (*n* = 5), or belly (*n* = 1)	18	Rarely	
Catheter dislodgement due to a rupture at the cannula-catheter intersection	1	Rarely	
Catheter dislodgement attributed to the absence of a clothing clip, especially when fixed on the belly and leg	1	Rarely	
Catheter dislodgement occurred due to accidentally screwing the cannula “button”	3	Rarely	
In exceptional cases, the catheter dislodged completely, involving both the cannula and catheter, such as when the catheter got caught in a car door or during leg use	3	Rarely	
Catheter dislodged at work, particularly when fixed in the belly and leg	1	Daily	
Catheter dislodged when the cannula is fitted to the leg	1	Weekly	
Reported experiencing catheter dislodgement without providing details of the circumstance	4	Monthly	

Diabetes Center Insulin Pump Ambulatory, Federal University of São Paulo (São Paulo, Brazil, 2021).

**Table 8:** Characterization of the type and frequency of “Catheter knotting” and proposals for educational actions.

Catheter knotting (catheter not dislodged) (*n* = 28)			
AEs	*n*	Frequency	Actions
Temporary knot with auto-resolution	8	Rarely	Patient Actions: For patients who experience unexplained hyperglycemic episodes, it is recommended to administer two corrections in “bolus” mode using the CSII. If blood glucose levels remain high despite these corrections, they should administer insulin with a pen and change the IIS. It is also important for patients to evaluate with the health team the factors that may have contributed to the situation to identify alternatives for improvement. In cases of unexplained hyperglycemia, after changing the IIS, patients should show the damaged catheter to the healthcare team for further evaluation. Healthcare Team/Educator Actions: The health team or diabetes educator is advised to check glucose monitoring downloads for patterns of sub-alarm occlusions, which can indicate issues with wear time or catheter function. They should also review the frequency of IIS changes and provide guidance if the current frequency is not recommended or if adjustments are needed. This helps ensure proper management and effectiveness of the insulin infusion therapy.
Knot during catheter pocket placement	2	Rarely	
Knot during sleep or sitting, affecting blood glucose	9	Rarely	
Catheter knotting (cause unknown)	7	Rarely	
Catheter knotting during sleep, impacting blood glucose	2	Weekly	
Reported experiencing this adverse event without providing details	3	Monthly	

Diabetes Center Insulin Pump Ambulatory, Federal University of São Paulo (São Paulo, Brazil, 2021).

**Table 9:** Characterization of the type and frequency of “Catheter bending” and proposals for educational actions.

Catheter bending (catheter not dislodged) (*n* = 44)			
AEs	*n*	Frequency	Actions
Sleeping or in daily living activities	11	Rarely	Patient Actions: Patients are recommended to change the IIS if they experience an occlusion alarm or if they encounter unexplained hyperglycemia that does not respond to a correction bolus. Additionally, patients should bring any removed catheter, used during the hyperglycemic episode, for evaluation by the healthcare team. Healthcare Team/Educator Actions: The healthcare team or diabetes educator should regularly review continuous glucose monitoring (CGM) sensor downloads to identify patterns of sub-alarm occlusions, as they may indicate potential issues with the CSII. It is also essential to review the frequency of available IIS options and ensure that they align with recommended guidelines, making necessary adjustments as needed. This helps ensure the proper functioning and effectiveness of insulin infusion therapy.
When the cannula is on the flank or leg	2	Rarely	
After animals bite the catheter, causing insulin flow interruption	3	Rarely	
When the catheter wire wraps around clothing or a clip involuntarily, turning whitish	5	Rarely	
When positioning the catheter in the bra	1	Rarely	
Reported experiencing this adverse event without providing details	19	Rarely	
During sleep	1	Monthly	
Catheter bending resulted in whitening in the line (no air on the line)	2	Monthly	

Diabetes Center Insulin Pump Ambulatory, Federal University of São Paulo (São Paulo, Brazil, 2021).

These findings highlight the potential risk of accidental catheter traction and the various circumstances in which it may occur. It is important to address this AE to minimize its occurrence and ensure the stability and effectiveness of the IIS.

- Occurrence of catheter knotting: this AE refers to the rare occurrence of a knot in the catheter of the IIS, with the following characteristics:
  − *N* = 8 patients experienced the AE, and in these instances, the catheter knotting dissolved spontaneously without any occlusion message or interruption in the insulin flow.
  − *N* = 2 reported experiencing this AE when the catheter was positioned in a pocket.
  − *N* = 9 patients experienced the AE while sleeping or sitting, which had negative repercussions on their blood glucose levels.
  − Additionally, *n* = 7 patients experienced this AE without providing any specific reason for the occurrence.
  − *N* = 2 patients reported experiencing this AE weekly while sleeping, and it had negative effects on their glycemic control.

− *N* = 3 patients reported experiencing this AE monthly, but no further details about the circumstances were provided.

It is important to note that the occurrence of an IIS catheter knot, although rare, can have implications for the functionality and effectiveness of the insulin infusion system. The negative impact on blood glucose control in certain situations emphasizes the importance of addressing and mitigating this AE to ensure optimal treatment outcomes.

- Occurrence of catheter bending: this AE has been reported by 36 patients and involves catheter bending, highlighting potential challenges and complications associated with its use. This AE manifests itself in a variety of circumstances as described below.
  − *N* = 11 patients experienced the AE while sleeping or during their daily activities.
  − *N* = 2 patients reported a bent catheter when it was positioned on the flank or leg.
  − *N* = 3 patients encountered the AE after animals bit the catheter, leading to interruption of insulin flow.
  − *N* = 5 patients described the catheter wire involuntarily getting wrapped around clothing or a clip, resulting in the catheter becoming whitish.

− *N* = 1 patient experienced the AE when positioning the catheter in the bra.

− Additionally, *n* = 14 patients reported experiencing this AE without providing further details about the circumstances.

− *N* = 1 patient reported this AE occurring monthly during sleep.

− *N* = 2 patients reported that catheter bending led to its whitening, but the circumstances were not specified.

The various situations mentioned highlight the need for careful handling and positioning of the catheter to minimize the occurrence of these AEs.

Insulin leakage at the catheter-reservoir intersection is another occurrence related to catheters. This event was not experienced by 103 out of the total number of patients and was rarely reported by 15 patients. The circumstances contributing to this issue included accidental traction at the connection point (reported by 1 patient), incorrect positioning of the catheter in the device (reported by 2 patients), improper passage of the catheter (reported by 2 patients), and incorrect threading of the reservoir cap (reported by 4 patients). These factors can lead to insulin leakage at the junction between the catheter and the reservoir, which must be addressed to ensure proper functioning of the IIS.

These incidents underscore the importance of proper handling and installation of the catheter and reservoir to prevent insulin leakage and ensure effective insulin infusion therapy. To address this issue, it is recommended that patients inspect the device, reservoir/cartridge, tubing, and IIS if they experience unexplained hyperglycemia symptoms. If any leakage is detected, the odor of insulin is present, or if a bolus correction is ineffective, the IIS should be changed.

During the initial training, healthcare teams or diabetes educators should familiarize the patient with the scent of insulin, enabling them to detect any potential leakage. They should also verify the patient’s technique when installing the IIS and closing the cartridge/reservoir to ensure proper installation and minimize the risk of insulin leakage. Proper education and training on IIS handling and installation, along with regular monitoring and immediate corrective actions, can help prevent and address insulin leakage at the catheter-reservoir intersection, ensuring the safety and efficacy of insulin infusion therapy.

#### 3.3.2 Cannula

Table 10 describes AEs related to the IIS cannula, along with proposed solutions to ensure patient clinical stability in response to these occurrences. In the study, 114 patients (96.7%) adhered to the recommended rotation of the cannula application site across five designated areas: 69% on the abdomen, 65% on the flank, 54% on the gluteus, 40% on the leg, and 37% on the arm. However, despite adherence to these guidelines, the incidence of Lipohypertrophy remained persistently high, reaching 64.4%.

**Table 10:** Characterization of the type and frequency of AEs related to IIS cannula and proposals for educational actions.

Adverse events	*n*	Actions
Cannula adhesive easily peels off the skin in hot weather	4	**Patient Actions:** Inspect the device, reservoir/cartridge, tubing, and IIS if experiencing unexplained hyperglycemia. Change the IIS if you observe leakage, detect an insulin odor, or find that attempted bolus correction is ineffective. Check for a lack of connection between the cannula/catheter and/or reservoir catheter, especially for Medtronic_®_ devices (Veo model), to detect insulin leakage. Perform proper skin preparation and hygiene before inserting the cannula. Clean the skin with water, soap, and alcohol. Use a protective film (e.g., Cavilon) to create a barrier and protect the skin from body fluids and perspiration. Apply deodorant spray to dry skin before inserting the cannula. Apply corticoid spray to mitigate the effects of skin irritation. **Healthcare Team/Educator Actions:** Continuously inspect the cannula insertion sites on the skin for potential Lipohypertrophy development. Evaluate the size of the cannula used and instruct the patient to change it if necessary. A very large cannula may reach the muscle and bend, while a smaller caliber cannula tends to be positioned intradermally. When the cannula bends, always recommend the use of the smaller caliber cannula. Ensure that the patient is familiar with the smell of insulin for early detection of leakage. Assess the patient’s proficiency in cannula insertion technique and cartridge/reservoir filling. Verify whether the patient is adequately using the cannula “applicator.”
Strong adhesion of the glue causing local itching	1	
Patch easily peels off the skin after being worn for more than 3 days	1	
Signs of allergies to the sticker, such as redness, pain, or changes in appearance	5	
Insulin leakage occurring beneath the cannula patch	27	
Insulin leakage resulting from a disconnection of the cannula catheter	2	
Continuous insulin leakage into the skin due to accidental cannula traction	2	
Skin or inside the cannula bleeding, with or without insulin flow obstruction.	51	
Bleeding on the skin due to accidental cannula traction	1	
Signs of skin inflammation	48	
Cellulite develops where the cannula is inserted into the leg	1	
Skin pain and bleeding occurring if the cannulas are bent during removal	7	
Reduced fluid absorption and action when triggering the insulin “bolus” mode	1	

Diabetes Center Insulin Pump Ambulatory, Federal University of São Paulo (São Paulo, Brazil, 2021).

While the present study did not specifically analyze the outcomes of AEs alone, we conducted a recent study on AEs associated with the use of CSIIs. This study aimed to propose a taxonomy based on prevalent occurrences experienced by patients with T1D (29). In total, 159 AEs were identified, which resulted in 60 device changes for various reasons, including issues with cannulas and catheters.

### 3.4 Detection of alarm emission and interruption of insulin flow in response to AE related to the IIS catheter

#### 3.4.1 Detection of interruptions in basal/bolus programming

Thirty-nine patients reported experiencing interruptions in basal/ bolus programming.

Sixty-five patients did not experience interruptions in basal/ bolus programming.

Six patients reported experiencing interruptions sometimes.

Two patients reported experiencing interruptions rarely. Six patients could not provide an answer.

#### 3.4.2 Emission of insulin flow occlusion alarm

Thirty-five patients reported receiving an insulin flow occlusion alarm. Fifty-nine patients did not receive this type of communication.

Eighteen patients reported receiving the alarm sometimes. Six patients reported receiving the alarm rarely.

Devices manufactured by Medtronic trigger the alarm only when the insulin flow is completely occluded. Roche devices are capable of generating alarms only for partial occlusion.

When the “no adm” alarm occurs in the “basal” programming mode, it signifies a more serious issue (e.g., a motor problem with a potential risk of interrupting the insulin flow). However, when this alarm appears in “bolus” mode, it may indicate a fold or displacement of the IIS (Table 11).

**Table 11:** Characterization of interruption of basal/bolus programming in response to AEs.

		Was an occlusion alarm issued?		
		**YES**	**SOMETIMES**	**NO**
Did the IIS accidental catheter traction, catheter knotting, and catheter bending AEs interrupt the device’s basal flow or bolus programming?	YES	“No admin” alert for when the cannula is bent during removal (2 occurrences) Suspected bubble in the IIS catheter or when approaching the end of insulin in the reservoir (3 occurrences) Catheter bending (1 occurrence) Catheter knotting (1 occurrence) The issue was only noticed with an increase in blood glucose, progressing to ketoacidosis. Changing the IIS catheter resolved it. “No admin” message is present (5 occurrences) The occlusion message appears when using a 6 mm catheter. Cannula bending is suspected and is confirmed by the tortuosity when removing it (2 occurrences) No comments (8 occurrences).	An occlusion alarm, even if the cannula is outside the body (1 occurrence) When blood comes up through the cannula (1 occurrence) The problem was only noticed with an increase in blood glucose. “Non-admin” alert appears (2 occurrences) When sleeping on stomach and obstructing insulin flow (1 occurrence) Soon after changing the IIS, the patient noticed difficulty in resuming the flow of insulin (1 occurrence) No comments (4 occurrences).	Suspected IIS catheter occlusion (1 occurrence) The patient only perceives the problem due to an increase in glycemia, without an occlusion message, despite flow interruption (1 occurrence) Despite IIS cannula bending when removed, neither flow interruption nor an alarm was identified (1 occurrence) No comments (5 occurrences).
	SOMETIMES	When bending the catheter momentarily for some reason or when contracting and relaxing the arm (1 occurrence)	Catheter bending (1 occurrence) When a bubble is suspected inside the IIS cannula (1 occurrence) Improper application of the cannula to the skin (when it reaches a blood vessel) (1 occurrence) When faced with the occlusion message, turn the device on and off or change the entire IIS to resolve the event (1 occurrence) No comments (2 occurrences).	The occlusion message appears only when the insulin in the reservoir is running low (1 occurrence) The patient only notices the problem due to an increase in blood glucose or the smell of insulin (3 occurrences) An alarm arises when the arm is sore or when there is local bleeding upon removing the cannula (1 occurrence) No comments (40 occurrences).
	NO	An alarm appears when the volume of insulin in the reservoir is about to run out or when there is a suspected IIS catheter obstruction (2 occurrences) Suspicion of catheter obstruction coinciding with a rise in blood glucose (1 occurrence) No comments (7 occurrences).	“No admin” alert when there is cannula bending and it shows tortuosity when withdrawing (2 occurrences) When there is a suspicion of a bubble in the catheter or when the amount of insulin in the reservoir is running low (1 occurrence) When there is catheter knotting, an interruption in the flow or an occlusion message was not observed, nor an increase in blood glucose. However, when the cannula bends, an error message appears, and blood glucose levels increase (1 occurrence) No comments (2 occurrences)	The occlusion message appears only when the insulin in the reservoir is running low (1 occurrence) The patient only notices the problem due to an increase in blood glucose or the insulin smell (3 occurrences) Alarm arises when the arm is sore; local bleeding is present when removing the cannula (1 occurrence) No comments (40 occurrences).

Diabetes Center Insulin Pump Ambulatory, Federal University of São Paulo (São Paulo, Brazil, 2021).

## 4 Discussion

Recent innovations in CSII therapy demonstrate a heightened awareness of issues such as unexplained hyperglycemia and occlusion in IIS, regardless of the presence of an alarm. Early detection and prompt correction of these problems are critical for minimizing risks in automated insulin administration. The most common AEs include:

- Accidental catheter traction
- Catheter knotting
- Catheter bending
- Issues related to cannula fixation
- Insulin leakage
- Bleeding episodes
- Skin problems

The occurrence of alarms and occlusions highlights the need for further investigation and improvements in detection and response mechanisms to enhance patient safety.

### 4.1 Characteristics of IIS cannulas and catheters, and IIS change frequency recommendations

The sizes of IIS cannulas and catheters available in the market vary among manufacturers, as detailed below.

**Table:** 

Medtronic^1^	Roche^1^
**Silhouette:** Flexible cannula: 17 mm Inserted at an angle of 30 to 45 degrees Use of the Silhouette-Serter™ applicator Catheters 60 cm or 90 cm	**Accu-Check® Flexlink:** Flexible cannula: 6, 8, and 10 mm Inserted at a 90-degree angle Use of the Accu-Check® Link Assist applicator Catheters 30, 60, 80, and 110 cm
**Quick-Set:** Flexible cannula: 6 mm or 9 mm Inserted at a 90-degree angle Use of the Quick-Serter™ applicator Catheters 60 cm or 90 cm	**Accu-Check® Tenderlink**: Flexible cannula: 13 or 17 mm Manually inserted at an angle ranging from 20 to 45 degrees Catheters 30, 60, 80, and 110 cm

^1^Source: Oliveira and Galves (30).

The Diabetes Center Insulin Pump Ambulatory, Federal University of São Paulo recommends changing the IIS cannula and catheter every 3 days, regardless of the brand of the device. This schedule is based on their experience and research, ensuring the safety and effectiveness of the CSII. The rationale behind this recommendation is that after 3 days, the cannula could be perceived as a foreign object by the body, which may lead to potential inflammatory processes in the skin. Additionally, there is a possibility of fluids adhering to the pores of the catheter material, potentially impacting its performance. The center ensures that an adequate supply of materials is provided for this specific periodicity.

Similarly, the Medtronic company shares the same recommendation and justification for changing the cannula and catheter every 3 days. On the other hand, Roche suggests a different approach. They recommend replacing the cannula every 3 days and the catheter every 6 days. This means that a single catheter can be used for two cannula changes. Manufacturers often provide guidelines to ensure the proper functioning of their products. It is worth noting that, irrespective of these recommendations, all patients receive the necessary supplies free of charge from the Brazilian government.

The government’s costs in managing DM, in comparison to the benefits, can be partially mitigated by reducing expenses associated with treating T1D-related complications, and studies show that these additional costs can be recouped within 3 years (31). Furthermore, adhering to the recommended change intervals helps prevent skin inflammation and potential declines in the performance of the IIS and injection site (23, 25, 32). This not only ensures the continued effectiveness of treatment but also promotes optimal glycemic control and minimizes long-term complications of DM, guaranteeing the safe use of CSII.

It is interesting to note that patient behavior sometimes differs from official guidelines provided by healthcare teams or manufacturers. This can be due to various reasons, including:

1. User experience: some patients may find that adhering to the manufacturer’s recommended frequencies works best for them in terms of comfort, convenience, or insulin delivery effectiveness. They might have experienced fewer issues or complications by following this schedule.
2. Perceived effectiveness: users may believe that adhering to the manufacturer’s recommendations leads to better glycemic control or fewer episodes of hypoglycemia or hyperglycemia.
3. Resistance to change: individuals can be resistant to change, especially if they have successfully followed a particular routine for an extended period. They might hesitate to adopt a new schedule recommended by their healthcare team.
4. Lack of awareness: patients may not be aware of the official guidelines provided by their healthcare team or may not fully understand the rationale behind those guidelines.

Regardless of the reasons, healthcare professionals must maintain open lines of communication with their patients. Regular discussions about treatment plans and ensuring that patients understand the potential benefits and risks associated with different approaches are essential. Additionally, patients should feel comfortable discussing their concerns and preferences with their healthcare providers. By working collaboratively, they can make informed decisions about their diabetes management. Ultimately, the goal is to ensure that patients achieve the best possible outcomes and quality of life while effectively and safely managing their condition. In this context, it is known that changing the catheter in a 2- to 3-day regimen is not an evidence-based practice, with most reports in the scientific literature coming from the manufacturers themselves (15, 23, 25).

A double-blind, randomized, crossover study conducted in the USA in 2010 aimed to evaluate the impact of non-adherence to the recommended change interval for IIS lines. The study compared the use of insulin aspart and lispro, and investigated the effects of extending the use of the infusion line beyond 48 h. The results showed that to maintain glycemic control it is crucial to change the IIS every 48 h. However, it was also observed that, in the short term, this loss of glycemic control had no significant impact on oxidative stress and glycation, which are markers of metabolic and vascular complications associated with DM (23).

These findings highlight the importance of regular adherence to the recommended change interval for IIS lines to maintain optimal glycemic control. While short-term non-adherence may not immediately result in significant metabolic and vascular complications, it is still essential to follow the recommended guidelines to ensure long- term T1D management and minimize the risk of complications.

In the 2010 study conducted by Schmid et al., which involved 24 patients and had two observation periods of 3 months each, the tolerability of 2-day use of IIS was compared to 4-day use. The findings of the study indicated that using the IIS for longer than 2–3 days increased treatment-related tolerability issues. Specifically, the number of catheter-related AEs was 290 with 2-day use compared to 495 with 4-day use.

Delaying the change of IIS beyond the recommended interval (48–72 h) can pose risks and lead to various complications. One concern is the increased risk of inflammation or infection at the infusion site. Prolonged use of the IIS can create a conducive environment for bacterial growth and may result in localized infections. Additionally, a delay in changing the IIS can erode the patient’s confidence in effectively managing AEs, as they may face more frequent complications.

Furthermore, progressive worsening of glycemic control may occur as a result of prolonged use of the IIS. Insulin absorption and delivery can be compromised, leading to fluctuations in blood glucose levels and difficulties in maintaining stable glycemic control. This can have negative implications for overall DM management and may require additional interventions to regain control.

Patients need to be aware of the recommended guidelines for IIS change and understand the potential risks associated with delaying the change. Regular and timely change of the IIS is crucial for minimizing complications, maintaining treatment efficacy, and ensuring optimal glycemic control. It is essential to consider these contexts when developing educational actions for patients and health educators in the management of DM. By addressing patient specific needs and concerns, personalized educational initiatives can guide the use of IIS cannulas and catheters best suited to their needs, emphasizing factors such as hygiene, potential AEs, and the importance of regular changes to ensure effective insulin delivery and well-being.

Medical devices, including CSII, are continually evolving, and updated guidelines may become available as more research and real-world experiences contribute to the knowledge base.

### 4.2 Alarm emission and interruption of insulin flow in response to catheter AEs

Risk management, as per the ISO 14971 standard, is essential for the certification and safe use of CSII. It generates requirements aimed at safeguarding against adverse effects on safety caused by physical component failures, development errors, and user errors (33). These devices increasingly rely on embedded software and communication mechanisms that classify them as information systems (34).

In this context, Requirements Engineering utilizes methods, techniques, and tools to establish a foundation for software development, ensuring the definition and analysis of security requirements (35). Therefore, the elicitation and specification of requirements are crucial to ensure that the intended system operates following the relevant needs and constraints. Inadequate security requirements can result in damages and losses, rendering the entire CSII system development process infeasible and potentially endangering human lives. In this context, considering that the CSII is a critical safety system, additional care is taken to ensure that the description of safety requirements is organized into the following hazard categories: operational, hardware, and software (36).

The detection of alarms and interruption of insulin flow as a response to AEs specifically related to the IIS catheter indicates that a significant number of patients encountered disruptions in their basal or bolus programming, resulting in occlusion alarms. However, it is worth noting that a considerable portion of patients did not experience such interruptions or receive corresponding alarms. Various clinical factors and individual responses to insulin therapy may contribute to the occurrence of occlusions. Therefore, regular monitoring and adherence to the recommended IIS change intervals remain essential in clinical practice to ensure optimal insulin delivery and reduce the risk of occlusions.

There is a notable concern regarding “silent occlusions” in IIS. “Silent occlusions” refer to occlusions that may go unnoticed because IIS systems rely on in-line pressure to detect flow and activate occlusion alarms. Consequently, this can lead to inadequate glucose control and delays in recognizing insulin failure, resulting in hyperglycemia, diabetic ketosis, or ketoacidosis (37).

To evaluate the difference in occlusion rates among rapid-acting insulin analogs, *in vitro* tests were performed for 5 days. These tests showed no differences between the analogs in the first 48 h at high (bolus) or low administration rates. However, after that, occlusion rates varied with the analog used, with a rate of 40.9% for insulin glulisine and 9.2% for insulin aspart. The authors concluded that early occlusions are uncommon and independent of insulin type and that infusion sets should be changed at least every 72 h (37).

### 4.3 Catheter and cannula AEs, and proposals for educational actions

A randomized trial conducted in 2014 (38) compared the function of a Teflon(_®_) catheter [Dupont(™), Wilmington, DE] or a steel catheter for CSII therapy in T1D and revealed some important findings on IIS-related AEs. In total, 13% of catheters were removed due to pain; 10% were accidentally tractioned out; 10% experienced erythema (redness or inflammation); 5% fell out due to loss of adhesion; and 4% were removed due to infection.

These AEs cover a variety of complications that can arise from using the IIS, including discomfort, accidental dislocation, local skin reactions, and infections. This emphasizes the importance of carefully monitoring and managing IIS to minimize complications and ensure the optimal functioning of insulin delivery systems. Regular assessment of the infusion site, proper insertion techniques, and adherence to recommended change schedules can help reduce the occurrence of AEs associated with IIS and enhance the overall effectiveness of insulin therapy.

In a 2010 German study, several AEs related to IIS were investigated. The most common AE was hyperglycemic events, accounting for 74.8% (615 AEs) of the reported events. Other AEs included erythema (redness), rash, pain, redness at the injection site, skin irritation, bleeding, and various less frequent events. Skin irritations were reported by the patients. Specifically, patients reported 33 injection site reactions for the 2-day usage time, while 59 injection site reactions were reported for the 4-day usage time. This suggests that longer use of the IIS was associated with a higher frequency of problems with the adhesive used to secure the set in place (39).

These findings highlight the potential for various AEs and complications associated with the use of IIS. Skin irritations, problems with adhesive, and cannula crimping are among the challenges that may arise. Regular monitoring of the infusion site, proper adhesive application, and adherence to recommended usage times can help mitigate these issues and enhance patient comfort and safety during insulin therapy.

The considerations mentioned by (20) concerning preparing and maintaining skin integrity during the use of IIS are as follows:

1. Hand washing before changing the catheter site: this is an important step to ensure cleanliness and reduce the risk of infection.
2. Opening the IIS package on a table or clean area: maintaining a clean environment during the process helps minimize contamination.
3. Cleaning the top of the insulin vial with alcohol: wiping the vial with alcohol before drawing insulin helps maintain sterility.
4. Cleaning the infusion site with prepackaged skin cleansing wipes or soap and water: thoroughly cleaning the skin at the infusion site helps remove dirt and bacteria. This can be done using the prepackaged skin cleansing wipes (preferably non-alcoholic) or soap and water.
5. Cleaning the skin in an outward spiral: when cleaning the skin, it is recommended to wipe in an outward spiral motion rather than back and forth. This helps prevent the introduction of contaminants into the infusion site.
6. Letting the site air dry: allowing the cleaned infusion site to air dry is preferred over blowing on it, as blowing can introduce bacteria from the breath.
7. Testing for dryness by touching the outside edge, not the center: when checking if the site is dry, it is advised to touch the outside edge of the site rather than the center. This minimizes the risk of introducing contaminants to the center of the site.

Following these considerations can contribute to maintaining skin integrity, reducing the risk of infection, and promoting the safe and effective use of IIS.

Unexplained hyperglycemia can pose a significant challenge to the success of insulin infusion therapy. In cases where unexplained glucose levels remain elevated (>250 mg/dL or 13.88 mmol/L) and do not decrease at least 2 h after a correction bolus, the recommendation is to change the IIS and reservoir with insulin from a new vial or to consider manual dosing. The patient should make an effort to identify the cause of IIS failures, such as dislocation, blockage, scar tissue, or leakage, and take note of the circumstances if possible. If glycemic control cannot be restored within a reasonable period if nausea and vomiting persist, or if the patient’s condition continues to worsen, emergency care may be necessary (20, 38).

When it comes to disconnections of the CSII for activities such as showering or swimming, it is recommended to check blood glucose levels both before and after the disconnection period. If the anticipated disconnection is expected to last for an hour or longer, it is commonly suggested that the patient switch to basal insulin by administering a bolus dose immediately before the disconnection. This approach should be repeated if the disconnection time is extended, with intermittent CSII reconnections, until the disconnection period has ended (20).

These guidelines aim to address specific situations related to IIS use and help manage challenges such as unexplained hyperglycemia and temporary pump disconnections. Following these recommendations can assist in maintaining glycemic control and ensuring patient safety during the use of insulin infusion therapy.

Indeed, when a patient experiences IIS failure, it is important for the healthcare team to thoroughly review the site preparation and insertion technique. They should inspect the site, skin, and anchor line for any abnormalities or issues. Blood glucose and continuous glucose monitoring data should be carefully examined to identify any unexplained hyperglycemia that may be related to the IIS failure. It is also valuable to help the patient reconstruct the circumstances surrounding the episode, as this can provide insights and enable the development of self-care skills to prevent or manage future occurrences.

To deal with such occurrences, we list here possible proposals to solve the problems:

- Providing comprehensive education and training to patients on proper handling and care of the IIS catheter to minimize accidental catheter traction, catheter knotting, and catheter bending.
- Enhancing communication between healthcare providers and patients to promptly identify and address issues.
- Improving the accuracy and effectiveness of insulin flow occlusion alarms to promptly detect and communicate potential obstructions.

When it comes to diabetes education for patients using CSII treatment, there is a need to enhance training strategies. This includes increasing the availability of training materials from device manufacturers and suppliers. Additionally, it is recommended that healthcare professionals conduct in-person visits shortly after initial patient training to assess equipment settings, IIS placement, and application techniques, making necessary adjustments as needed.

By enhancing the quality of education and training materials, providing personalized support, and placing emphasis on ongoing maintenance, healthcare professionals can empower patients to effectively manage their treatment and optimize their insulin therapy. This approach ensures that patients who have difficulty identifying occlusion issues receive the appropriate guidance and assistance they need.

A study by Deeb et al. (40) aimed to evaluate the impact of targeted education on improving competence in solving alarm problems. They found that verbal and written instructions on alert solutions were well-received by patients, resulting in significant reductions in warnings and errors. Another review study conducted by Minicucci (16) highlighted that the safety and effectiveness of using CSII systems are strongly influenced by the level of diabetes education.

The educational approach directed toward patients who use CSII by the multi-professional team at the study site has proven highly beneficial for enhancing patients’ competence and response to diabetes management. Several noteworthy features of this approach in the Diabetes Center Insulin Pump Ambulatory program are as follows:

- Multidisciplinary team: the presence of a multidisciplinary healthcare team on-site is crucial for addressing the diverse needs of patients. This team includes psychologists, nutritionists, physical educators, nurses, insulin pump educators, and doctors. This holistic approach not only addresses the medical aspect but also focuses on the overall well-being of the patient.
- Educational initiatives: prioritizing diabetes education and proper device usage is essential for patients to comprehend their condition and the benefits of insulin therapy. The education provided by the Diabetes Center Insulin Pump Ambulatory team professionals equips patients with the knowledge and skills to manage their health effectively.
- Initial training: the initial training provided is tailored for newly diagnosed patients. This ensures that they acquire the necessary skills to safely and effectively use the device right from the start.
- Ongoing guidance: continual education and periodic guidance are essential for maintaining the quality of care over time and keeping patients informed about best practices in diabetes and insulin device management. The text messaging approach proves to be an effective way to maintain regular contact with patients.
- Age segmentation: segmenting patients into different age groups for educational guidance is a unique approach at the site. This recognizes that needs and challenges vary across different life stages, allowing the healthcare team to customize their guidance for each group. Patients are grouped into the following age categories for educational support: 0–3 years (group 1); 4–8 years (group 2); 8–10 years (group 3); 11–13 years (group 4); 13–15 years (group 5); 16 to 18 (group 6); 18–20 (group 7); 21–25 years (group 8); and over 30 years (group 10).

## 5 Conclusion

Educating patients about common causes of IIS failure is crucial for ensuring their safety and optimizing CSII therapy. The research results can serve as a guide to improve the understanding of possible AEs associated with IIS failures, thus enhancing patient education and treatment.

Standardized guidelines for preventing and diagnosing IIS issues are essential for maximizing the benefits of CSII therapy. While the optimal time to switch to IIS is still debated in the literature, recent studies and advancements in IIS science are shedding light on this matter. It is important to address problems such as unexplained hyperglycemia and occlusions when discussing IIS-related issues.

Patients should be trained and encouraged to follow the manufacturer’s recommendation regarding changing the cannula and catheter every 2–3 days. While the cost of frequent catheter changes may be a consideration, it should not be the determining factor for neglecting necessary changes.

The key findings from the research include:

- Serious situations (AEs) occur in cases where the occlusion alarm is not activated but the insulin flow is interrupted, or in less worrisome situations with alarm activation, without interruption of the insulin flow.
- Most individuals adhere to the recommended frequency of changing the IIS cannula and catheter within the recommended time.
- Individuals who change the cannula and catheter on different days tend to keep the catheter longer than recommended but usually change the cannula within 3 days.
- Prolonged catheter use is associated with an increased frequency of catheter-related AEs.
- Catheter-related accidental traction and catheter bending typically occur during everyday activities, while cannula-related issues directly affect blood glucose levels.
- AEs related to the IIS cannula often lead to skin problems.

By incorporating these findings into patient education and treatment protocols, healthcare professionals can enhance patient safety, improve glycemic control, and minimize the occurrence of AEs related to IIS failures. It is important to note that the main limitation of this study is its reliance on data from a single center, which restricts the generalizability of the findings. Therefore, conducting extended research through randomized trials or a prospective multicenter study to investigate clinical outcomes would provide valuable insights.

## Data availability statement

The original contributions presented in the study are included in the article/supplementary material, further inquiries can be directed to the corresponding author.

## Ethics statement

The studies involving humans were approved by Research Ethics Committee – University Federal of São Paulo. The studies were conducted in accordance with the local legislation and institutional requirements. Written informed consent for participation in this study was provided by the participants’ legal guardians/next of kin.

## Author contributions

AN: Conceptualization, Formal analysis, Investigation, Methodology, Writing – review & editing, Writing – original draft. LM: Conceptualization, Formal analysis, Investigation, Methodology, Writing – review & editing, Funding acquisition, Project administration, Resources, Supervision, Validation, Visualization. MG: Conceptualization, Investigation, Project administration, Resources, Supervision, Validation, Writing – review & editing, Writing – original draft. PP: Formal analysis, Writing – original draft. TO: Formal analysis, Writing – original draft. AM: Formal analysis, Writing – original draft. SD: Formal analysis, Writing – original draft. DC: Formal analysis, Writing – original draft. SA: Formal analysis, Funding acquisition, Resources, Writing – original draft. FT: Formal analysis, Conceptualization, Investigation, Methodology, Validation, Writing – original draft, Writing – review & editing, Visualization. TC: Conceptualization, Formal analysis, Investigation, Methodology, Validation, Writing – original draft, Writing – review & editing, Funding acquisition, Project administration, Resources, Supervision, Visualization.
